# Disentangling Refractive Index Contributions in Transient Absorption Spectroscopy of Two-Dimensional Halide Perovskites

**DOI:** 10.1021/acs.jpclett.5c02744

**Published:** 2025-10-23

**Authors:** Xian Wei Chua, Yorrick Boeije, Taeheon Kang, Arjun Ashoka, Shabnum Maqbool, Akshay Rao, Samuel D. Stranks

**Affiliations:** † Department of Chemical Engineering and Biotechnology, 2152 University of Cambridge, Philippa Fawcett Drive, Cambridge CB3 0AS, United Kingdom; ‡ Cavendish Laboratory, Department of Physics, 2152 University of Cambridge, JJ Thomson Avenue, Cambridge CB3 0HE, United Kingdom

## Abstract

Two-dimensional (2D) halide perovskites are intensely researched for emerging light-emitting and -harvesting technologies due to their outstanding optoelectronic properties, strong quantum confinement, and enhanced ambient stability over their three-dimensional counterparts. A powerful technique for understanding their excited-state dynamics is transient absorption (TA) (pump–probe) spectroscopy. However, the interpretation can be complicated by simultaneous reflectivity changes arising from their high refractive index. Here, we adopt a Kramers–Kronig constrained variational analysis to disentangle these effects, as demonstrated for the prototypical Ruddlesden–Popper 2D perovskite phenylethylammonium lead iodide (PEA_2_PbI_4_). We show that photoinduced changes in the real and imaginary parts of the complex dielectric function can be of similar magnitude, but find that reflectivity effects do not imprint significantly on the TA spectra or kinetics. Our work clarifies the role of refractive index contributions in the TA spectroscopy of 2D perovskites, reconciles literature views, and provides confidence in the analysis of TA data for these emerging semiconductors.

## Graphical abstract

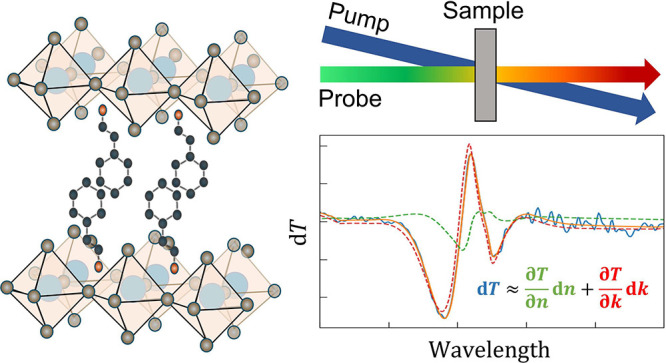

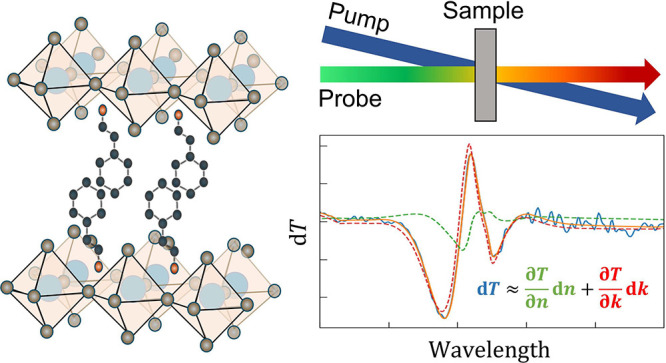

Two-dimensional (2D) metal halide perovskites have been intensely researched semiconductors over the past decade.^1,2^ Their outstanding optoelectronic properties have put them at the vanguard of emerging light-emitting^3,4^ and light-harvesting^5–9^ technologies. They consist of layers of metal halide octahedra quantum wells intercalated with organic chains.^10^ Due to strong quantum confinement within the inorganic slabs,^11^ and poor dielectric screening from the surrounding ligands, the exciton binding energies are typically significant and lie between 200 and 300 meV.^1,12^ Additionally, the hydrophobic and bulky organic cation ligands act as insulating barriers that lead to enhanced ambient stability against oxygen and moisture.^13^

Unlike dilute molecular systems where changes in reflectivity are often negligible in TA spectroscopy,^14^ the higher refractive index of inorganic semiconductors like halide perovskites^15,16^ can introduce strong photoinduced refractive index changes that occur simultaneously with absorption changes. This can affect the changes in transmission and obscure the true photoinduced spectra.^17–20^ The transmission is further modulated by reflections from the front and back interfaces of the thin film, due to refractive index mismatches. However, there have been differing views on the extent of these refractive index contributions to the measured TA spectra for halide perovskites. For example, the above-gap photoinduced absorption (PIA) feature in CH_3_NH_3_PbI_3_ has been assigned to changes in the imaginary part of the refractive index due to free carrier injection.^21^ It is proposed that transient reflectivity changes lead to both a significant sharpening and enhancement of the ground-state bleach (GSB), while also generating an above-gap negative transmission spectral feature.^21^ Due to the presence of organic spacer layers, the refractive index of 2D perovskites can be lower than that of 3D perovskites,^22^ since the organic layers reduce the overall electronic polarizability and dielectric constant. Nonetheless, the refractive index of 2D perovskites is still non-negligible, ∼1.3–3.2 for (PEA)_2_PbI_4_ thin films, as measured from ellipsometry.^23,24^ For (PEA)_2_PbI_4_, previous studies have attributed the two PIA bands to photoinduced refractive index changes, in addition to linewidth broadening of the exciton transition.^25^ On the contrary, other works have suggested that refractive index effects are minor. For example, based on thin-film and nanocrystal measurements of CsPbI_3_ and CH_3_NH_3_PbI_3_ 3D perovskites, Ghosh et al. found that only small corrections were needed to correct for photoinduced reflectivity effects. They concluded that none of the key spectral features in TA were exclusively or strongly influenced by changes in sample reflectivity.^14^

Given conflicting interpretations, it is important to quantitatively assess the magnitude of these effects to accurately interpret TA data.^26–28^ Approaches in literature have included simultaneous differential transmission and reflection measurements,^21^ multiangle reflectance analysis,^29^ pump–probe ellipsometry,^30^ and frequency domain interferometry.^17,31,32^ Here, we aim to disentangle these effects using a Kramers–Kronig constrained variational analysis,^33–35^ applied to the prototypical Ruddlesden–Popper 2D perovskite (PEA)_2_PbI_4_, where PEA is phenylethylammonium. We focus on PEA_2_PbI_4_ as a representative system because it was the first 2D organic–inorganic metal halide perovskite incorporating a conjugated organic group (a phenyl ring), and has since been extensively studied.^36–40^ We find that photoinduced changes in the real and imaginary parts of the complex dielectric function can be of similar magnitude in these 2D systems, but reflectivity effects do not imprint significantly on the TA spectra or kinetics. Our work clarifies the extent of refractive index contributions in TA spectroscopy of 2D perovskites, a result consistent with 3D perovskite analogues,^14^ and reconciles different perspectives in the literature.

We investigate spin-coated thin films of polycrystalline (PEA)_2_PbI_4_ (thickness ∼ 100 nm) prepared by a single-step spin-coating of stoichiometric (1:2 PbI_2_:PEAI ratio) precursor solution in DMF/DMSO (4:1) on UV-ozone-treated glass substrates (see Methods for details). X-ray diffraction confirms their high crystallinity with no detectable crystalline impurity phases (Figure S1a). The films exhibit a strong excitonic absorption peak at 516 nm (Figure S1b), while the photoluminescence (PL) is centered at 522 nm (Figure S1c). A biexponential fit to the PL decay yields an effective lifetime of 3.0 ns (Figure S1d).

We performed TA spectroscopy, which measures the differential transmission Δ*T*/*T* of a broadband optical probe as a function of time delay after photoexcitation with an ultrafast pump. Here, *T* is the probe transmission without the pump, and Δ*T* is the difference in transmission induced by the pump. We excite the films above the bandgap at 3.10 eV (400 nm) at a range of fluences from 0.24 μJ cm^–2^ to 2.71 μJ cm^–2^ (corresponding charge carrier densities are listed in Table S1). The TA spectra at 1–10 ps are shown in Figure 1a. In general, these spectra reflect a complex interplay of various effects, including spectral shifts and linewidth variations.^41^ These effects typically occur simultaneously, making TA data nontrivial to interpret.^41^

**Figure 1:**
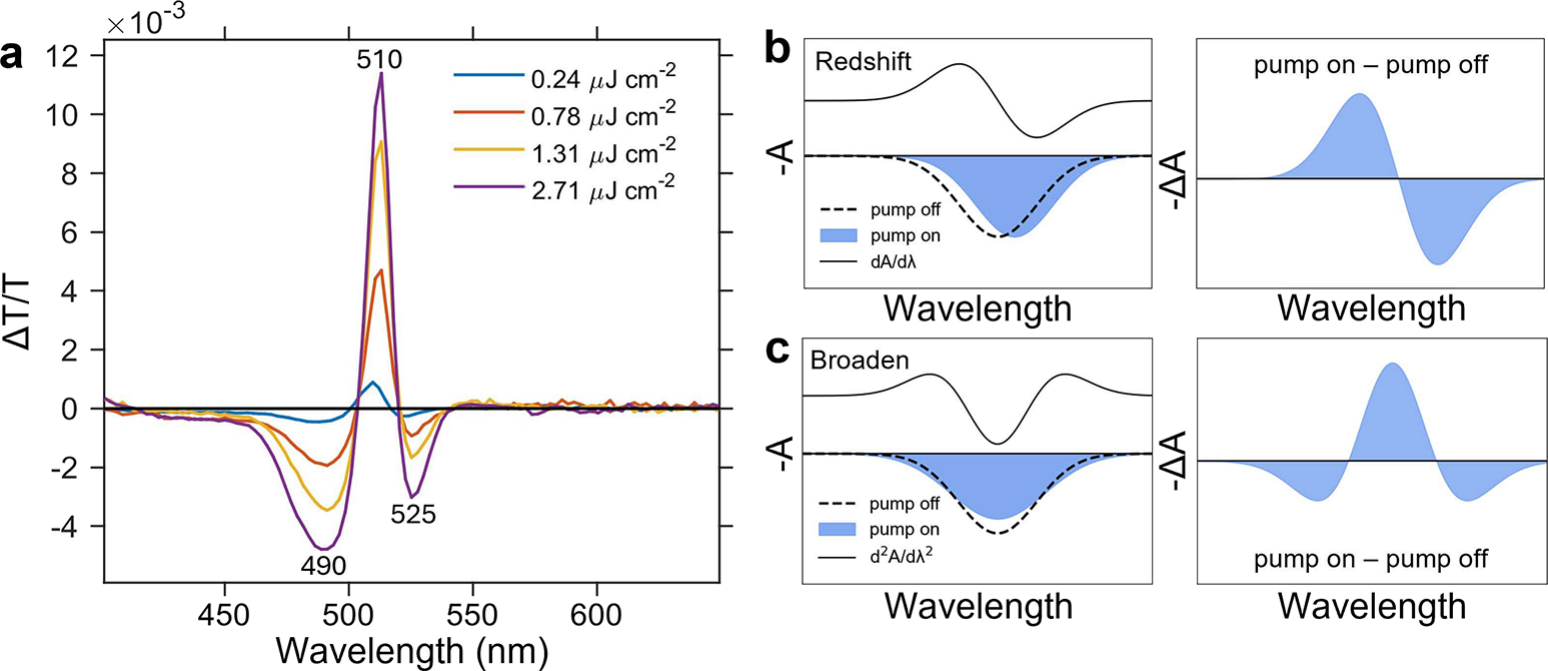
(a) Fluence-dependent Δ*T*/*T* spectra at 1–10 ps for the (PEA)_2_PbI_4_ thin film, with the pulse fluence stated in the legend (excitation at 400 nm). (b, c) Schematic illustration of the effects of (b) spectral redshift and (c) linewidth broadening on −Δ*A*, compared with d*A*/dλ and d^2^ *A*/dλ^2^.

The positive Δ*T*/*T* feature at ∼508–510 nm corresponds to the ground-state bleach (GSB), arising from phase-space filling upon photoexcitation. Pauli exclusion prevents the excitation of already-occupied states, leading to a reduction in the oscillator strength of the excitonic transition.^42^ Notably, the positive Δ*T*/*T* feature also resembles the first derivative of the absorbance with respect to wavelength (Figures 1b and S2a), which may also indicate a spectral redshift. The negative Δ*T*/*T* features at ∼487–490 nm and ∼523–525 nm have been attributed to photoinduced refractive index changes, in addition to linewidth or collisional broadening of the exciton transition.^25,43^ The latter is supported by the resemblance of the negative Δ*T*/*T* subgap feature to the negative of the second derivative of the absorbance, d^2^ *A*/dλ^2^ (Figures 1c and S2a).^41^ Alternative explanations include the presence of another *J*-state,^43^ trap-state filling from the relaxation of above-gap excitations, exciton–phonon coupling,^45^ or a polaronic origin.^46^ The above-gap feature may also result from photoinduced absorption (PIA) to higher levels during hot carrier cooling.^46^ A rigorous assessment of refractive index contributions is essential for accurately interpreting the TA spectra and understanding the photophysics of these materials.^21^

We adopt a Kramers–Kronig constrained variational analysis,^31,34,47^ applied to the complex dielectric function ε­(ω) = ε_1_(ω) + iε_2_(ω), which characterizes a material’s response to an external electric field. We have recently demonstrated the use of this method to retrieve the full changes in dielectric function from broadband pump–probe spectroscopy, which were applied to thin film semiconductors including CsPbBr_3_ perovskite and pentacene.^34^ In TA spectroscopy, the imaginary part of the dielectric function is of particular interest, as it provides insights into the true photoexcited carrier dynamics in terms of the joint density of states and the oscillator strength of the transition. For example, ground state bleaching results in a decreased joint density of states at the original transition energy and consequently a negative Δε_2_. On the other hand, photoinduced absorption leads to an increased joint density of states with new transitions and produces a positive Δε_2_ (ref^34^). The idea of our analysis is to express ε­(ω) as the sum of a formula-defined function ε_mod_(ω) and a variational function ε_var_(ω), both of which satisfy the Kramers–Kronig relations.^33^ We do not assign a physical meaning to individual oscillators as particular electronic excitations or species. Instead, we use this approach to extract the underlying dielectric function from the measured TA spectra in a manner independent of any fitting model.^34,47^

Briefly, we first approximate ε_mod_(ω) as the sum of several Drude–Lorentz oscillators to capture the major features of the experimental spectra:^48^

1 $$\varepsilon_{\mathrm{mod}}\left(\omega \right)=\varepsilon_{\infty }+\overset{N_{\mathrm{mod}}}{\underset{k=1}{\sum }}\frac{\omega_{p,k}^{2}}{\omega_{0,k}^{2}-\omega^{2}-i\omega \gamma_{k}}$$

where each oscillator has three parameters: the oscillator frequency ω_0_, the plasma frequency ω_p_, and the linewidth γ. ε_∞_ represents the contribution from higher-frequency oscillators. The variational dielectric function ε_var_(ω) is then constructed as a linear superposition of triangular functions ε^Δ^ (Figure S3), which are anchored at equidistant energy spacings:

2 $$\varepsilon_{\mathrm{var}}\left(\omega \right)=\overset{N_{\mathrm{var}}}{\underset{k=1}{\sum }}A_{i}\varepsilon_{i}^{\Delta }\left(\omega \right)$$

where the amplitude coefficients *A*_*i*_ are free parameters.

We apply this analysis to the differential transmission spectra d*T*, which are derived by multiplying the measured Δ*T*/*T* with the steady-state transmission spectrum *T*, at individual time points. To separate the relative contributions of the real and imaginary parts to d*T*, we perform a first-order Taylor expansion, assuming small perturbations in *n*, *k*, ε_1_, and ε_2_:

3 $$dT=\frac{\partial T}{\partial n}dn+\frac{\partial T}{\partial k}dk$$

4 $$dT=\frac{\partial T}{\partial \varepsilon_{1}}d\varepsilon_{1}+\frac{\partial T}{\partial \varepsilon_{2}}d\varepsilon_{2}$$

The real and imaginary contributions to the TA spectra at 1.8 ps (a representative time point after hot carrier cooling), at a moderate fluence of 1.31 μJ/cm^2^, represented in the above equations by the first and second terms respectively, are illustrated in Figure 2 (see Figure S4 for the fitted steady-state *T*, *n*, and *k* and Figure S5 for plots of d*n*, d*k*, dε_1_, and dε_2_). Strikingly, we observe that while both d*k* and d*n* (or equivalently dε_1_ and dε_2_) can be similar in magnitude, the contribution to the d*T* spectra of d*n* (or dε_1_), when modulated by the first derivatives of *T*, is overall minor. This is because the derivatives of *T* with respect to *k* (or ε_2_) are several times larger than the derivatives with respect to *n* (or ε_1_) (Figure S6–7). In fact, the refractive index contributions only play a larger role in the TA spectra near the zero-crossings of the GSB and PIA features. We can therefore exclude the case where the PIA bands in the TA spectra of (PEA)_2_PbI_4_ arise purely from photoinduced refractive index changes.

**Figure 2:**
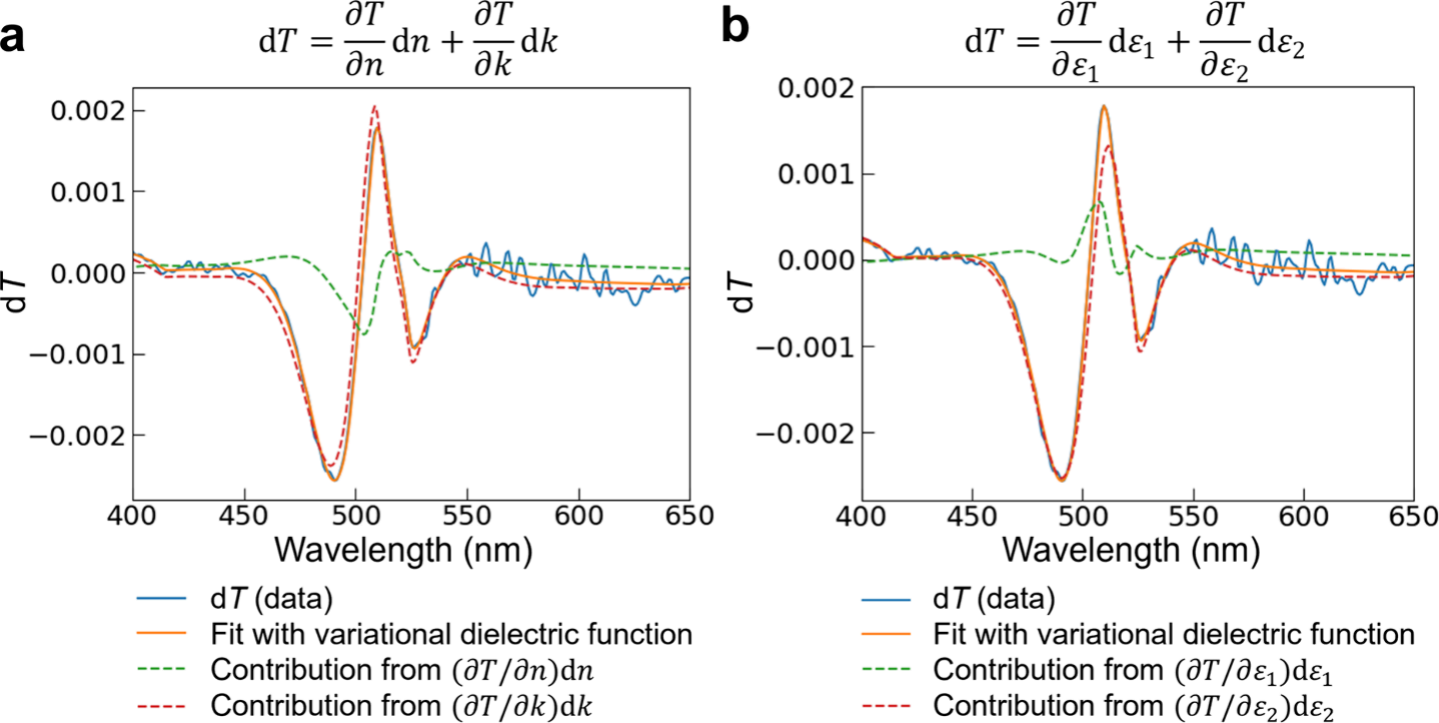
Contributions to d*T* from the real and imaginary parts of the (a) complex refractive index (based on eq 3) and (b) complex dielectric constant (based on eq 4) at 1.8 ps for the spin-coated (PEA)_2_PbI_4_ film (excitation at 400 nm, fluence 1.31 μJ/cm^2^). The contributions from the real parts are small and do not imprint significantly on the overall TA spectra.

To further examine these effects, we perform a one-to-one comparison of the maps, spectra and kinetics of d*T*/*T*, d*k*, and d*n* over time in Figure 3. The maps in Figure 3a provide an overall visual representation of the spectral evolution with increasing pump–probe delays, with the spectra at selected time slices shown in Figure 3b. Importantly, we observe that the spectra of d*k* closely resemble those of d*T*/*T*. Moreover, the kinetics of d*k* also mirror closely those of d*T*/*T*, across all the main spectral features, including the GSB (Figure 3d), but especially the above-gap PIA (Figure 3c) and subgap PIA (Figure 3e). These results clearly show that the TA spectra and kinetics of (PEA)_2_PbI_4_ can be interpreted relatively reliably using the original d*T*/*T* data, with only small modifications caused by photoinduced reflection effects.

**Figure 3:**
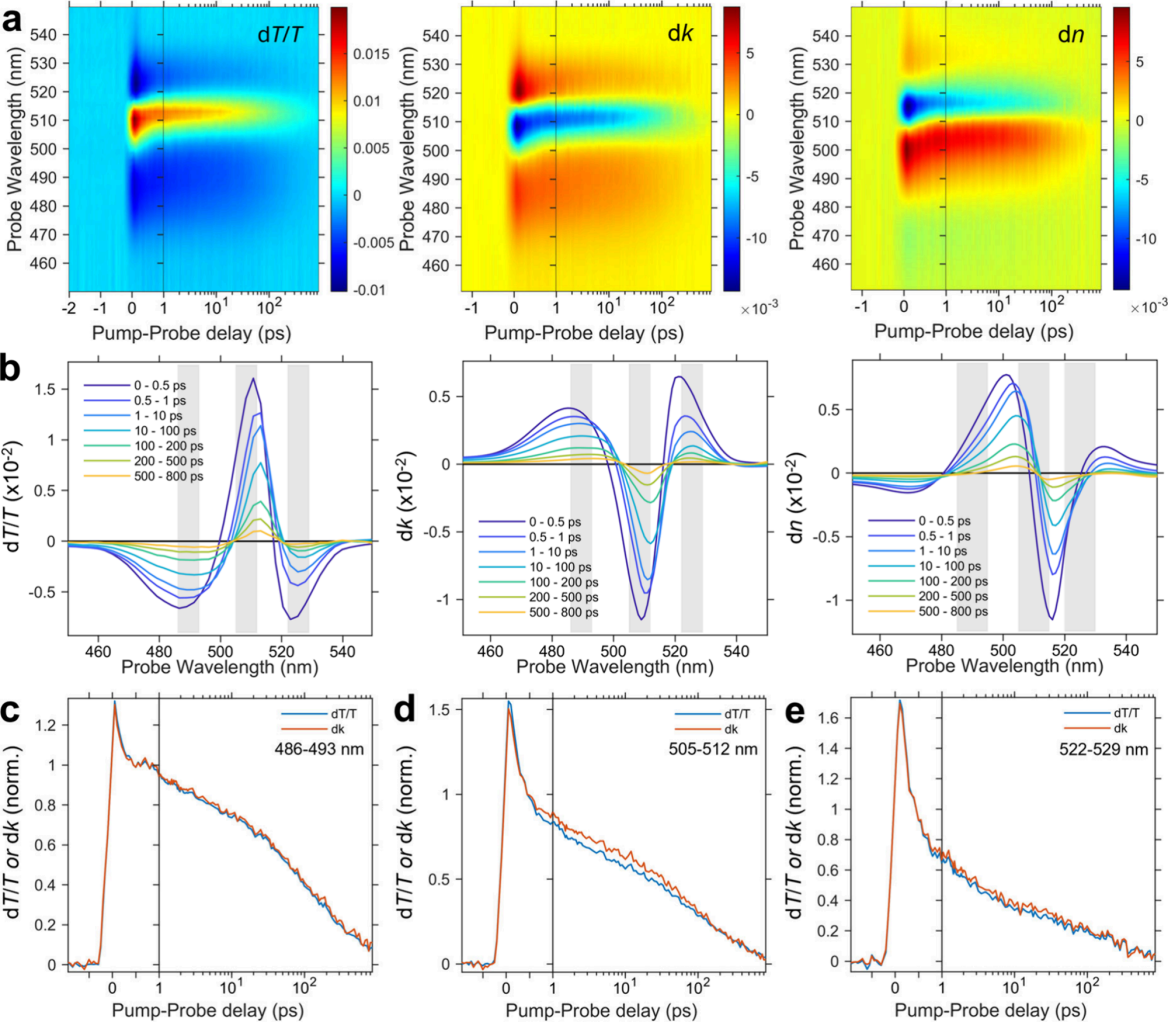
Comparison of the (a) maps and (b) spectra of d*T*/*T*, d*k*, and d*n* for the spin-coated (PEA)_2_PbI_4_ film, excited at 400 nm with a fluence of 2.71 μJ/cm^2^, as a function of pump–probe time delay. (c–e) Comparison of kinetics, normalized at 0.44 ps (after hot carrier cooling), for (c) PIA 486–493 nm, (d) GSB 505–512 nm, and (e) PIA 522–529 nm between d*k* and d*T*/*T*.

Our results provide a resolution to the apparent contradictory views in the literature regarding the significance of photoinduced refractive index effects. While these effects can be large in magnitude, similar to photoinduced absorption changes (Figure 3b), they do not significantly imprint on the Δ*T*/*T* spectra, which are primarily dominated by photoinduced absorption changes. Therefore, we clarify that although refractive index effects may be substantial, their impact on the TA spectra is negligible.

To showcase the generality of the results, we applied the same analysis procedure to thin films of (PEA)_2_PbI_4_ prepared by thermal coevaporation of PbI_2_ and PEAI (Figures S2 and S5–S10; see Methods). We performed TA spectroscopy on the evaporated film at identical fluences as the spin-coated films (Figure S8) and find similar conclusions that refractive index effects on the TA spectra and kinetics remain insignificant in these systems (Figure S9, cf. Figure 2). This example demonstrates the utility of the Kramers–Kronig analysis in disentangling refractive index effects from TA spectra, allowing a more accurate interpretation of the underlying photoexcited dynamics.

In conclusion, we have disentangled photoinduced refractive index contributions to the TA spectroscopy of the prototypical Ruddlesden–Popper 2D perovskite, (PEA)_2_PbI_4_, by adopting a Kramers–Kronig constrained variational analysis. Our results show that while changes in the real part of the refractive index can be significant and comparable in magnitude to photoinduced absorption effects, these reflectivity effects do not imprint significantly on the TA spectra. The differential transmission d*T*/*T* spectra and kinetics can be interpreted with only minor contributions from refractive index effects. Our results extend earlier findings on 3D perovskite analogues based on direct measurements of photoinduced refractive index contributions,^17,49^ as well as other experimental,^14^ analytical^18,34,47^ and computational^50^ approaches.

We applied our analysis to both spin-coated and evaporated films, which showcases the generality of the approach to investigating films prepared by different processing techniques. Our work provides important insights to reconcile the differing views on the magnitude of refractive index effects in the TA spectroscopy of 2D perovskites, and reinforces the utility of a Kramers–Kronig based analysis approach to effectively decouple refractive index contributions. Furthermore, the work also complements previous results on 3D perovskites employing other approaches, and provides confidence in the interpretation of d*T*/*T* spectra and kinetics from the measured TA data.

## Methods

### Materials

Lead iodide (PbI_2_) and phenethylammonium iodide (PEAI) were purchased from TCI. *N*,*N*-Dimethylformamide (anhydrous, 99.8%, 227056) and dimethyl sulfoxide (anhydrous, >99.9%, 276855) were purchased from Sigma-Aldrich. All materials were used as received.

### Sample Preparation of Spin-Coated (PEA)_2_PbI_4_ Thin Film

Scribed glass (1 in. × 1 in.) was cleaned in the sonication bath with the following steps for 15 min each: DI water, acetone and isopropanol. The cleaned substrate was transferred to UV-ozone chamber (UVC1014, NanoBioAnalytics) for another 15 min post-treatment. To make the perovskite solution, the precursors (PbI_2_ and FAI) were mixed in one vial in a 2:1 molar ratio to achieve 1 M concentration in DMF/DMSO (4:1) for the composition (PEA)_2_PbI_4_. The perovskite solution was prepared in a N_2_-filled glovebox (H_2_O and O_2_ below 1 ppm) and stirred for 3 h before use. 120 μL perovskite solution was spread on the glass and spun at 3000 rpm for 40 s. The samples were moved to a hotplate for post-annealing at 100 °C for 10 min.

### Sample Preparation of Evaporated (PEA)_2_PbI_4_ Thin Film

The same perovskite composition was thermally evaporated in a dual-source evaporation of the same precursors with identical postannealing procedure above. The deposition was controlled by quartz crystal microbalance (QCM) on each source in an evaporation chamber (Creaphys) enclosed with a cold wall. The rates were calibrated by iteratively adjusting the tooling factor. PEAI evaporation rate was maintained at excess, deviating from the stoichiometric nominal PbI_2_:PEAI ratio of 1:2. The temperature range for the evaporation of PEAI was between 160 and 190 °C, and that for PbI_2_ was between 200 and 220 °C.

### Steady-State Absorption and Photoluminescence Spectroscopy

Absorption spectra were measured using an Agilent Cary 7000 UV–vis–NIR spectrophotometer. Photoluminescence spectra were measured on an Edinburgh Instruments FLS1000 fluorimeter with a 450 W continuous xenon arc lamp.

### X-ray Diffraction

XRD measurements were performed using a Bruker X-ray D8 Advance diffractometer with Cu Kα radiation (λ = 1.5406 Å). Diffractograms were collected within an angular range of 5° ≤ 2θ ≤ 50° in steps of 0.02453°.

### Time-Correlated Single Photon Counting

TCSPC plots were measured on an Edinburgh Instruments FLS1000 fluorimeter equipped with a 450 W continuous xenon arc lamp. A picosecond pulsed diode laser at 404 nm (HPL-405) was used (repetition rate of 5 MHz), which was connected to the spectrometer using a coupling flange.

### Picosecond to Nanosecond Transient Absorption Spectroscopy

The 800 nm output of a Ti:sapphire amplifier system (Spectra Physics Solstice Ace) operating at 1 kHz and generating ∼100 fs pulses was split into the pump and probe beam paths. The ultraviolet–visible broadband beam (400–700 nm) was generated by focusing the 800 nm fundamental beam onto a CaF_2_ crystal (Eksma Optics, 5 mm) connected to a digital motion controller (Mercury C-863 DC Motor Controller) after passing through a mechanical delay stage (Thorlabs DDS300-E/M). The same output was used to generate a pump wavelength of 400 nm by second-harmonic generation (SHG) through a β-barium borate crystal. The pump was blocked by a chopper wheel rotating at 500 Hz. The transmitted pulses were collected with a monochrome line scan camera (JAI SW-4000M-PMCL, spectrograph: Andor Shamrock SR-163) with collected data fed straight into the computer.

### Kramers–Kronig Constrained Variational Analysis

In the complex dielectric function ε­(ω) = ε_1_(ω)+ iε_2_(ω), the real and imaginary parts are not independent but obey the Kramers–Kronig relations:^28,31,51–53^

M1 $$\varepsilon_{1}\left(\omega \right)=1+\frac{2}{\pi }P\int_{0}^{\infty }\frac{{\omega^{\prime }\varepsilon }_{2}\left(\omega^{\prime }\right)}{{\omega^{\prime }}^{2}-\omega^{2}}\;d\omega^{\prime }$$

M2 $$\varepsilon_{2}\left(\omega \right)=-\frac{2\omega }{\pi }P\int_{0}^{\infty }\frac{\varepsilon_{1}\left(\omega^{\prime }\right)-1}{{\omega^{\prime }}^{2}-\omega^{2}}\;d\omega^{\prime }$$

where ω is real and *P* represents the principal value integral:

M3 $$P\int_{0}^{\infty }d\omega^{\prime }\equiv \underset{\delta \to 0}{\lim}\left(\int_{0}^{\omega -\delta }d\omega^{\prime }+\int_{\omega +\delta }^{\infty }d\omega^{\prime }\right)$$

The Kramers–Kronig relations follow causality (light absorption occurs after light interacts with the medium),^54^ and a similar relation holds between *n* and *k* of the complex refractive index.^32,55^ We express ε­(ω) as the sum of a formula-defined function ε_mod_(ω) and a variational function ε_var_(ω), both of which satisfy the Kramers–Kronig relations (see eqs 1 and 2):^33^

M4 $$\varepsilon_{\mathrm{total}}\left(\omega \right)=\varepsilon_{\mathrm{mod}}\left(\omega \right)+\varepsilon_{\mathrm{var}}\left(\omega \right)$$

We use the Levenberg–Marquardt algorithm for the fitting of oscillators by minimizing the sum of squares of the errors between the model and data. The algorithm is a hybrid technique combining the gradient descent and Gauss–Newton methods when parameters are far from and close to the optimal value, respectively, converging to the optimal solution.^56–59^ An example of the improvement in the fit to d*T* after the variational analysis is illustrated at 1.8 ps for the evaporated and spin-coated films at a fluence of 1.31 μJ/cm^2^ (Figure S10).

We use the Fresnel equations for a bifacial thin film to model the power transmission coefficient *T*(ε_1_, ε_2_) = |(1 – *r* ^2^)*t*|^2^, where *r* and *t* are the complex Fresnel reflection and transmission amplitude coefficients:^33^

M5 $$r=\frac{1-\sqrt{\varepsilon }}{1+\sqrt{\varepsilon }}$$

$$t=e^{i\frac{\omega }{c}d\sqrt{\varepsilon }}$$ M6

where *d* is the film thickness. We use these equations to calculate the derivatives $\frac{\partial T\left(\varepsilon_{1},\varepsilon_{2}\right)}{\partial \varepsilon_{1}}$ and $\frac{\partial T\left(\varepsilon_{1},\varepsilon_{2}\right)}{\partial \varepsilon_{2}}$ analytically,^34^ and similarly for $\frac{\partial T\left(n,k\right)}{\partial n}$ and $\frac{\partial T\left(n,k\right)}{\partial k}$ (see the Supplementary Note).

## Supplementary Material



## Data Availability

The data that support the findings of this study are available in Apollo, the University of Cambridge Repository, at https://doi.org/10.17863/CAM.122281.

## Notes

Additional TA spectra and KK analysis, including for the evaporated film (PDF)
