# Supplementary material for: Disentangling Refractive Index Contributions in Transient Absorption Spectroscopy of Two-Dimensional Halide Perovskites
Source: J Phys Chem Lett. 2025 Oct 23;16(43):11308–15. doi: 10.1021/acs.jpclett.5c02744 (PMC12581162; doi:10.1021/acs.jpclett.5c02744)
Supplement: Supplementary file 1 [file jz5c02744_si_001.pdf]

Supporting Information

Disentangling Refractive Index Contributions in Transient Absorption Spectroscopy of Two-Dimensional Halide Perovskites

Xian Wei Chua[1,2], Yorrick Boeije[1,2], Taeheon Kang[1], Arjun Ashoka[2], Shabnum Maqbool[1,2], Akshay Rao*[,2], Samuel D. Stranks*[,1,2]

[1] Department of Chemical Engineering and Biotechnology, University of Cambridge, Philippa Fawcett Drive, Cambridge CB3 0AS, United Kingdom.

[2] Cavendish Laboratory, Department of Physics, University of Cambridge, JJ Thomson Avenue, Cambridge CB3 0HE, United Kingdom.

*Corresponding authors: ar525@cam.ac.uk; sds65@cam.ac.uk

	Fluence 0.24 μJ cm^{-2}	Fluence 1.31 μJ cm^{-2}	Fluence 2.71 μJ cm^{-2}
n_0	3.16×10^{11} cm^{-2}	1.72×10^{12} cm^{-2}	3.56×10^{12} cm^{-2}

Table S1: Photoexcited carrier densities per unit area n_0 for the spin-coated $(PEA)_2PbI_4$ film at various fluences. The absorption at the 400 nm pump wavelength is 0.654. The film thickness is approximately 100 nm.

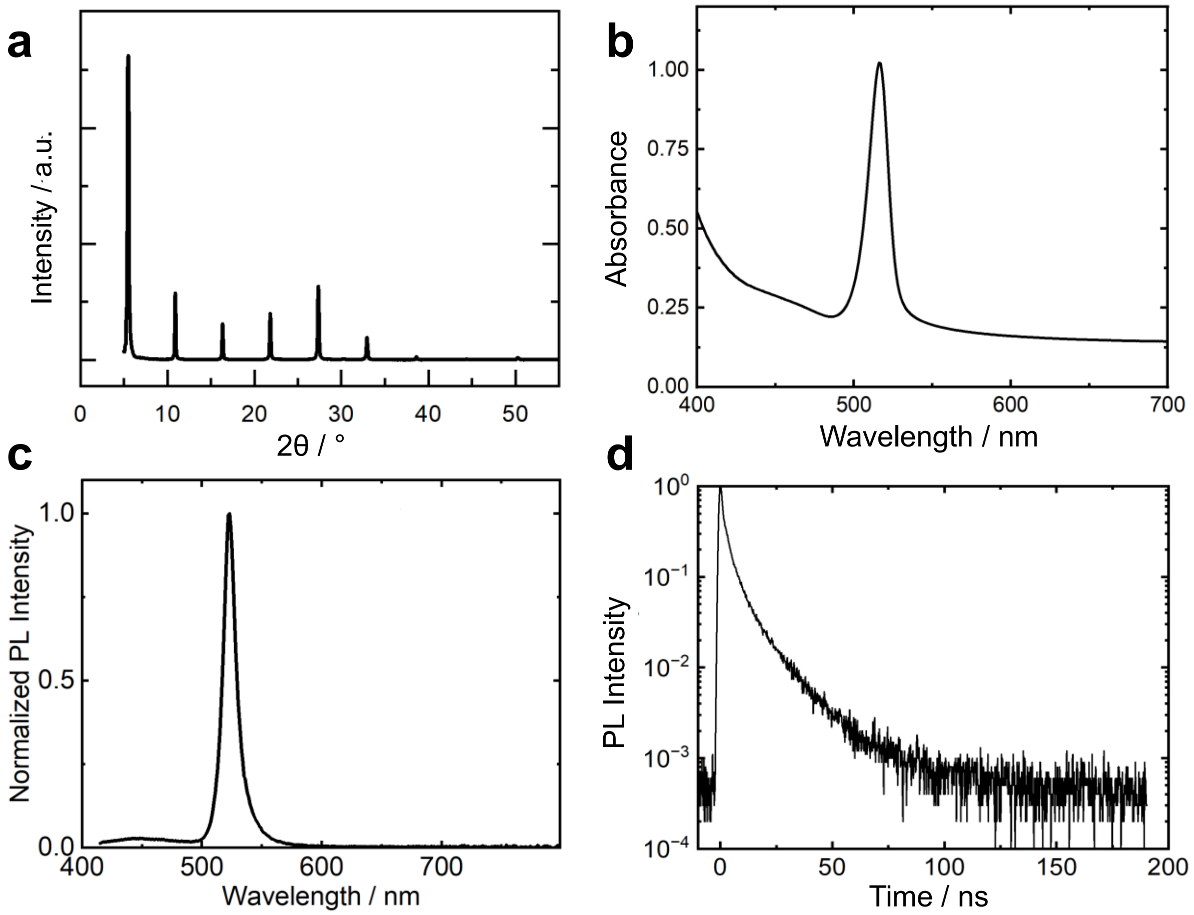

Figure S1: Structural and steady-state optical characterisation of the spin-coated $(PEA)_2PbI_4$ polycrystalline thin film, investigated by TA spectroscopy. (a) X-ray diffraction pattern using Cu-K_α radiation (λ = 1.5418Å). The diffraction peaks correspond to the {00l} family of crystallographic planes. (b) Steady-state absorbance and (c) photoluminescence spectra (excited at 400 nm). (d) Time-resolved photoluminescence decay excited at 405 nm at a fluence of ~0.1 μJ cm^{-2}.

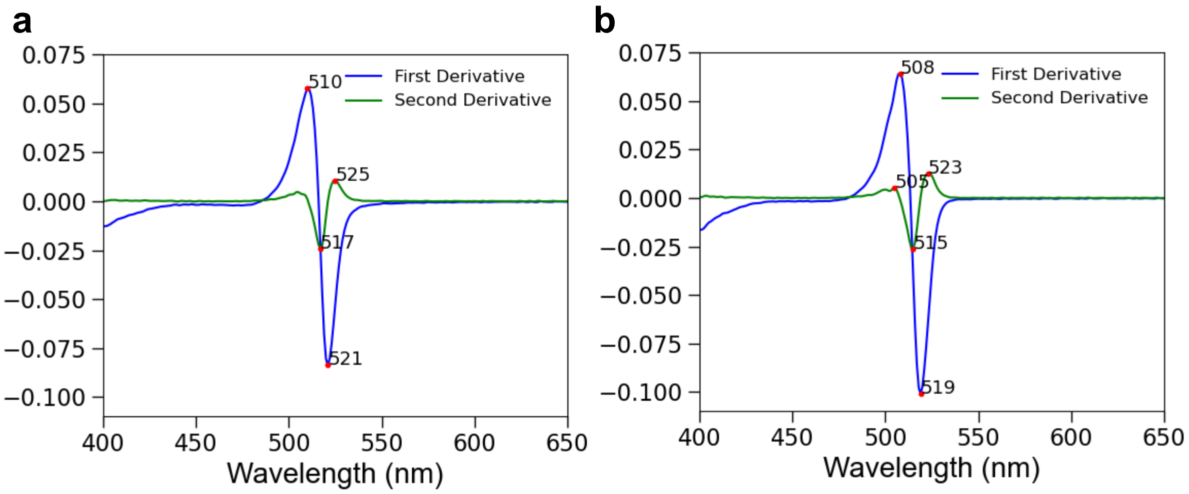

Figure S2: First and second derivatives of the absorbance with respect to wavelength (dA/dλ and d^2A/dλ^2, respectively) for the (a) spin-coated and (b) evaporated $(PEA)_2PbI_4$ films.

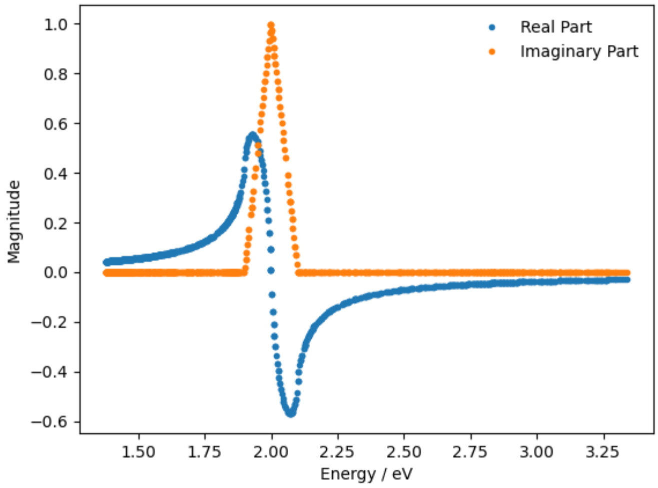

Figure S3: Example of a triangular function ε^{Δ} centered at 2.00 eV, illustrating the real and imaginary parts.

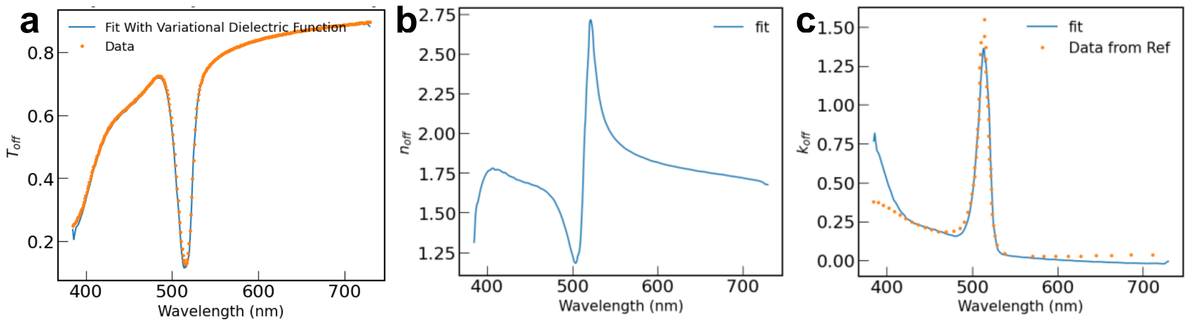

Figure S4: Fits to the steady-state (a) transmission function T_{off} using the variational dielectric function, along with the fitted (b) refractive index n_{off} and (c) extinction coefficient k_{off}. The extinction coefficient data from reference [1] is also digitised and overlaid in (c) for comparison. The steady-state fits were constructed from 4 Drude-Lorentz oscillators and 70 triangular oscillators.

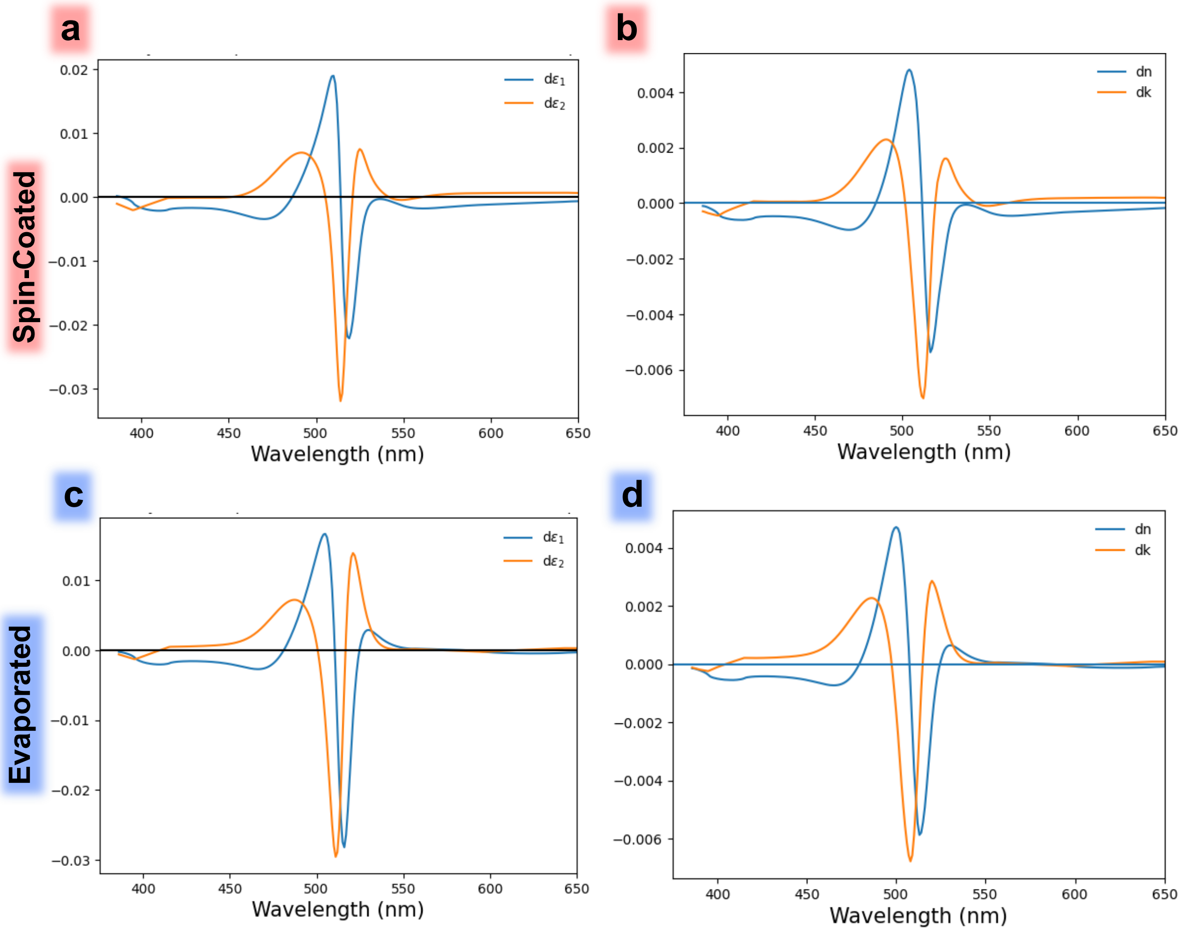

Figure S5: Comparison of the magnitude of changes in the real and imaginary parts of the (a) complex dielectric constant and (b) complex refractive index, at 1.8 ps for the spin-coated $(PEA)_2PbI_4$ film (fluence 1.31 μJ/cm^2). (c-d) Data for the evaporated $(PEA)_2PbI_4$ film.

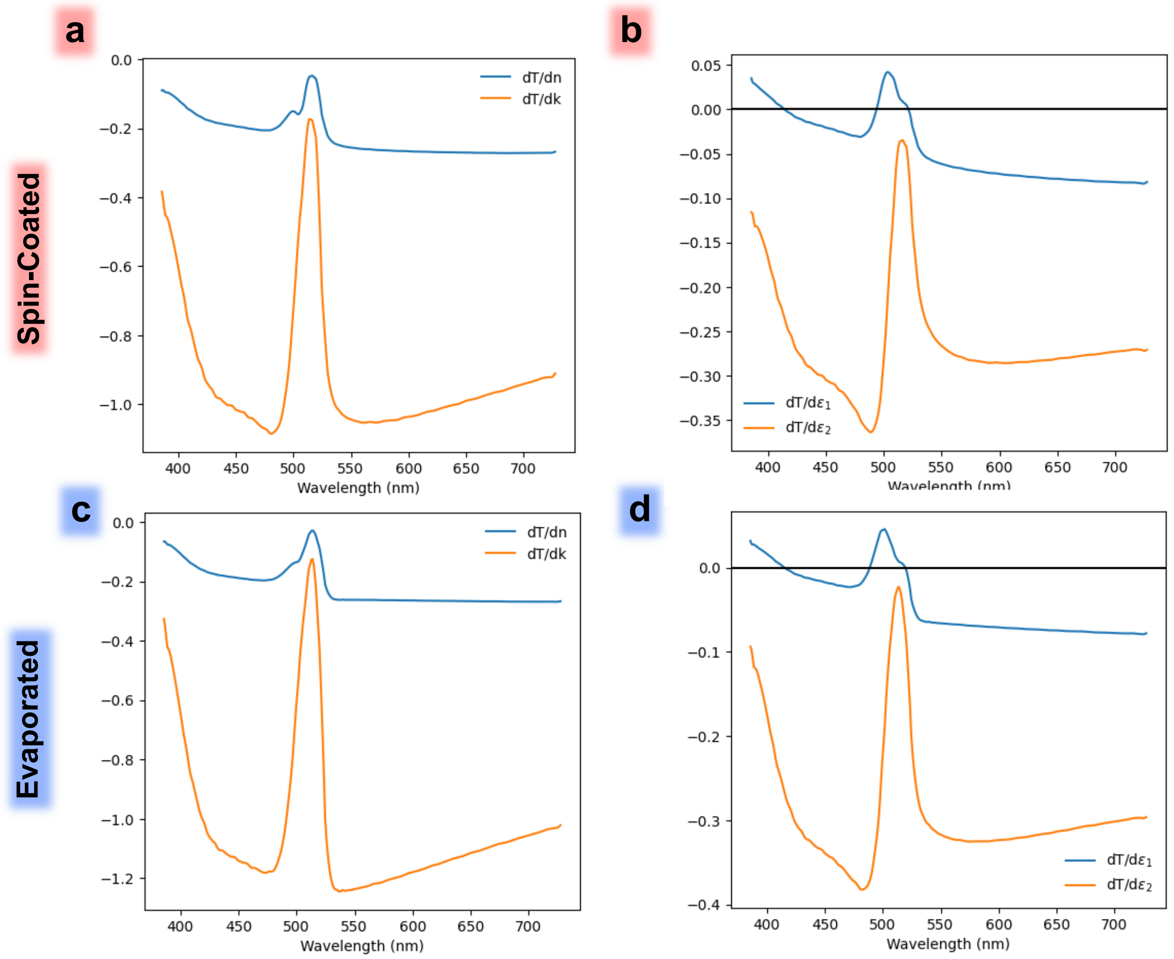

Figure S6: Comparison of the derivatives (a) $\frac{\partial T}{\partial n}$, $\frac{\partial T}{\partial k}$ and (b) $\frac{\partial T}{\partial \varepsilon_1}$, $\frac{\partial T}{\partial \varepsilon_2}$, at 1.8 ps for the spin-coated $(PEA)_2PbI_4$ film (fluence 1.31 μJ/cm^2). (c-d) Data for the evaporated $(PEA)_2PbI_4$ film.

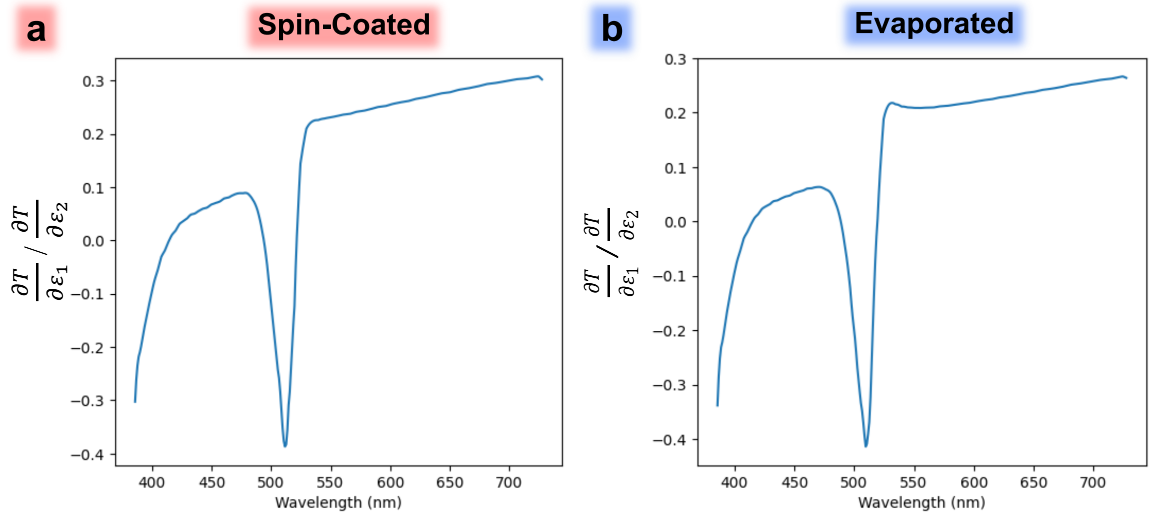

Figure S7: Ratio of $\frac{\partial T}{\partial \varepsilon_1}$ to $\frac{\partial T}{\partial \varepsilon_2}$ at 1.8 ps for the (a) spin-coated and (b) evaporated $(PEA)_2PbI_4$ films (fluence 1.31 μJ/cm^2).

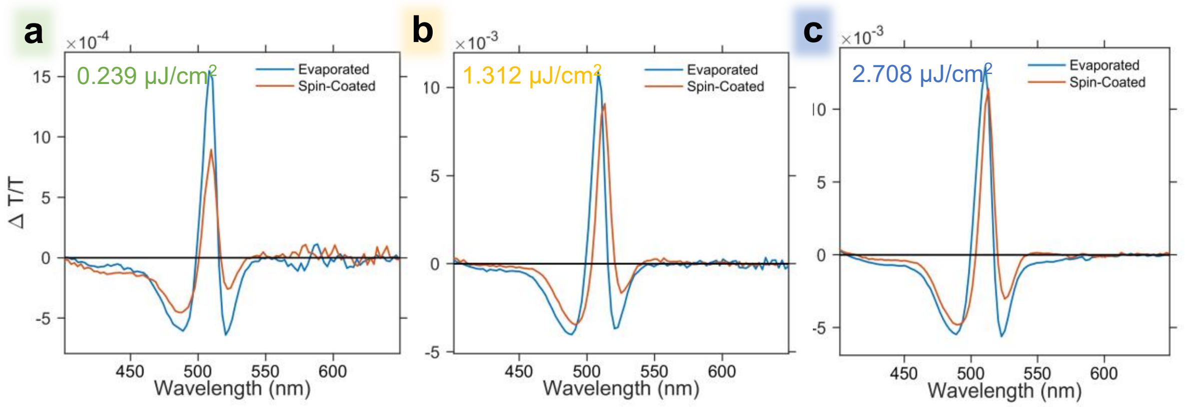

Figure S8: Comparison of ΔT/T spectra (1 to 10 ps) between evaporated and spin-coated $(PEA)_2PbI_4$ films, at three fluences: (a) 0.24 μJ/cm^2, (b) 1.31 μJ/cm^2, and (c) 2.71 μJ/cm^2.

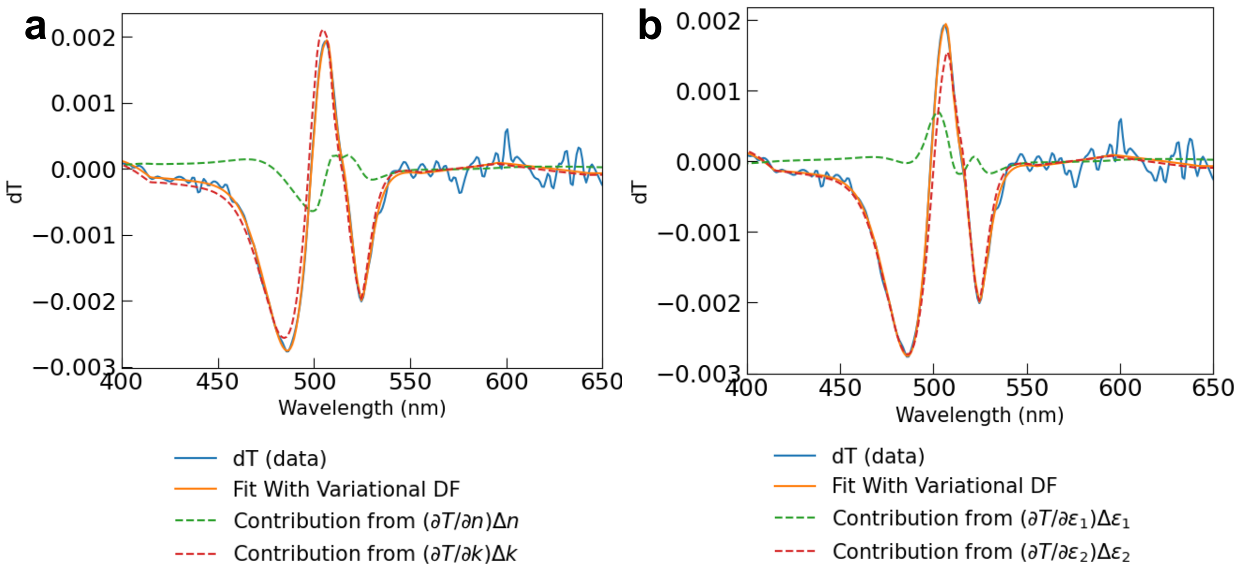

Figure S9: Contribution to dT by the real and imaginary parts of the (a) complex refractive index and (b) complex dielectric constant, at 1.8 ps for the evaporated $(PEA)_2PbI_4$ film (fluence 1.31 μJ/cm^2). The contribution by the real parts is small and does not imprint significantly on the overall TA spectra.

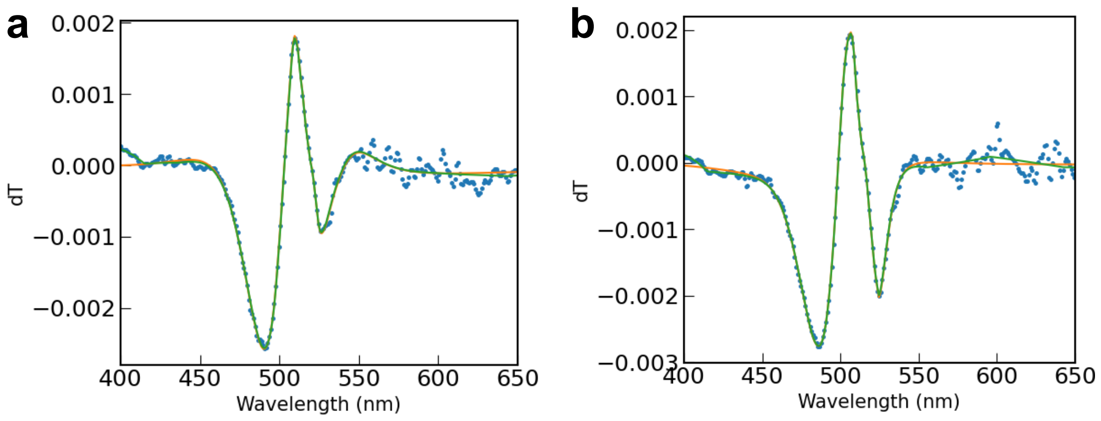

Figure S10: Fits to dT (data in blue) before (orange) and after (green) the variational analysis, at 1.8 ps for (a) spin-coated and (b) evaporated $(PEA)_2PbI_4$ films (fluence 1.31 μJ/cm^2), showing an improvement after adding a variational dielectric function made from triangular oscillators. The fits to dT were constructed from 6 Drude-Lorentz oscillators and 10 triangular oscillators.

Supplementary Note

We list the analytical expressions for the derivatives $\frac{\partial T}{\partial \epsilon_{\mathrm{re}}}$ and $\frac{\partial T}{\partial \epsilon_{\mathrm{im}}}$ of the power transmission coefficient $T(\epsilon_{\mathrm{re}}, \epsilon_{\mathrm{im}})$ here:

$$\frac{\partial T}{\partial \epsilon_{\mathrm{re}}} = \frac{v \cdot du_1 - u \cdot dv_1}{v^2} \quad (S1)$$

$$\frac{\partial T}{\partial \epsilon_{\mathrm{im}}} = \frac{v \cdot du_2 - u \cdot dv_2}{v^2} \quad (S2)$$

where:

$$u(w, \epsilon_{\mathrm{re}}, \epsilon_{\mathrm{im}}, d) = 16\sqrt{{\epsilon_{\mathrm{re}}}^2 + {\epsilon_{\mathrm{im}}}^2} \cdot \exp(-2a(\epsilon_{\mathrm{re}}^2 + \epsilon_{\mathrm{im}}^2)^{1/4} \sin\left(\frac{1}{2}\arctan\left(\frac{\epsilon_{\mathrm{im}}}{\epsilon_{\mathrm{re}}}\right)\right)) \quad (S3)$$

$$\begin{aligned} v(w, \epsilon_{\mathrm{re}}, \epsilon_{\mathrm{im}}, d) &= \left(\sqrt{{\epsilon_{\mathrm{re}}}^2 + {\epsilon_{\mathrm{im}}}^2} \cdot \left(\sin^2\left(\frac{1}{2}\arctan\left(\frac{\epsilon_{\mathrm{im}}}{\epsilon_{\mathrm{re}}}\right)\right)\right)\right. \\ &\left. + \left(1 + \sqrt[4]{{\epsilon_{\mathrm{re}}}^2 + {\epsilon_{\mathrm{im}}}^2} \cdot \cos\left(\frac{1}{2}\arctan\left(\frac{\epsilon_{\mathrm{im}}}{\epsilon_{\mathrm{re}}}\right)\right)\right)^2\right)^2 \quad (S4) \end{aligned}$$

$$\begin{aligned} du_1(w, \epsilon_{\mathrm{re}}, \epsilon_{\mathrm{im}}, d) &= \frac{1}{(\epsilon_{\mathrm{re}}^2 + \epsilon_{\mathrm{im}}^2)^{3/2}} \cdot \exp\left(-2a(\epsilon_{\mathrm{re}}^2 + \epsilon_{\mathrm{im}}^2)^{1/4} \sin\left(\frac{1}{2}\arctan\left(\frac{\epsilon_{\mathrm{im}}}{\epsilon_{\mathrm{re}}}\right)\right)\right) \\ &\cdot \left(16a(\epsilon_{\mathrm{re}}^2 + \epsilon_{\mathrm{im}}^2)^{1/4}(\epsilon_{\mathrm{re}}^2\epsilon_{\mathrm{im}} + \epsilon_{\mathrm{im}}^3) \cos\left(\frac{1}{2}\arctan\left(\frac{\epsilon_{\mathrm{im}}}{\epsilon_{\mathrm{re}}}\right)\right)\right. \\ &- 16a(\epsilon_{\mathrm{re}}^2 + \epsilon_{\mathrm{im}}^2)^{1/4}(\epsilon_{\mathrm{re}}^3 + \epsilon_{\mathrm{im}}^2\epsilon_{\mathrm{re}}) \sin\left(\frac{1}{2}\arctan\left(\frac{\epsilon_{\mathrm{im}}}{\epsilon_{\mathrm{re}}}\right)\right) + 16\epsilon_{\mathrm{re}}^3 \\ &\left. + 16\epsilon_{\mathrm{re}}\epsilon_{\mathrm{im}}^2\right) \quad (S5) \end{aligned}$$

$$\begin{aligned} du_2(w, \epsilon_{\mathrm{re}}, \epsilon_{\mathrm{im}}, d) &= \frac{1}{(\epsilon_{\mathrm{re}}^2 + \epsilon_{\mathrm{im}}^2)^{3/2}} \times \exp\left(-2a(\epsilon_{\mathrm{re}}^2 + \epsilon_{\mathrm{im}}^2)^{1/4} \sin\left(\frac{1}{2}\arctan\left(\frac{\epsilon_{\mathrm{im}}}{\epsilon_{\mathrm{re}}}\right)\right)\right) \\ &\cdot \left(-16a(\epsilon_{\mathrm{re}}^2 + \epsilon_{\mathrm{im}}^2)^{1/4}(\epsilon_{\mathrm{re}}^2\epsilon_{\mathrm{im}} + \epsilon_{\mathrm{im}}^3) \sin\left(\frac{1}{2}\arctan\left(\frac{\epsilon_{\mathrm{im}}}{\epsilon_{\mathrm{re}}}\right)\right)\right. \\ &- 16a(\epsilon_{\mathrm{re}}^2 + \epsilon_{\mathrm{im}}^2)^{1/4}(\epsilon_{\mathrm{re}}^3 + \epsilon_{\mathrm{im}}^2\epsilon_{\mathrm{re}}) \cos\left(\frac{1}{2}\arctan\left(\frac{\epsilon_{\mathrm{im}}}{\epsilon_{\mathrm{re}}}\right)\right) + 16\epsilon_{\mathrm{im}}\epsilon_{\mathrm{re}}^2 \\ &\left. + 16\epsilon_{\mathrm{im}}^3\right) \quad (S6) \end{aligned}$$

$$dv_1(w, \epsilon_{\mathrm{re}}, \epsilon_{\mathrm{im}}, d)$$

$$= \frac{2}{(\epsilon_{\mathrm{re}}^2 + \epsilon_{\mathrm{im}}^2)^{\frac{7}{4}}}$$

$$\cdot \left\{ \sqrt{\epsilon_{\mathrm{re}}^2 + \epsilon_{\mathrm{im}}^2} \cdot \sin^2\left(\frac{\arctan\left(\frac{\epsilon_{\mathrm{im}}}{\epsilon_{\mathrm{re}}}\right)}{2}\right) + \sqrt{\epsilon_{\mathrm{re}}^2 + \epsilon_{\mathrm{im}}^2} \cdot \cos^2\left(\frac{\arctan\left(\frac{\epsilon_{\mathrm{im}}}{\epsilon_{\mathrm{re}}}\right)}{2}\right) + 2 \right.$$

$$\left. \cdot (\epsilon_{\mathrm{re}}^2 + \epsilon_{\mathrm{im}}^2)^{\frac{1}{4}} \cdot \cos\left(\frac{\arctan\left(\frac{\epsilon_{\mathrm{im}}}{\epsilon_{\mathrm{re}}}\right)}{2}\right) + 1 \right\}$$

$$\cdot \left\{ \left(\epsilon_{\mathrm{re}}^3 + \epsilon_{\mathrm{im}}^2 \epsilon_{\mathrm{re}}\right) \cdot \cos\left(\frac{\arctan\left(\frac{\epsilon_{\mathrm{im}}}{\epsilon_{\mathrm{re}}}\right)}{2}\right) + (\epsilon_{\mathrm{re}}^2 + \epsilon_{\mathrm{im}}^2)^{\frac{1}{4}} \cdot \left(\epsilon_{\mathrm{re}}^3 + \epsilon_{\mathrm{im}}^2 \epsilon_{re}\right) \right.$$

$$\cdot \cos^2\left(\frac{\arctan\left(\frac{\epsilon_{\mathrm{im}}}{\epsilon_{\mathrm{re}}}\right)}{2}\right) + \sin\left(\frac{\arctan\left(\frac{\epsilon_{\mathrm{im}}}{\epsilon_{\mathrm{re}}}\right)}{2}\right)$$

$$\left. \cdot \left[\epsilon_{\mathrm{re}}^2 \epsilon_{\mathrm{im}} + (\epsilon_{\mathrm{re}}^2 + \epsilon_{\mathrm{im}}^2)^{\frac{1}{4}} \cdot (\epsilon_{\mathrm{re}}^3 + \epsilon_{\mathrm{im}}^2 \epsilon_{\mathrm{re}}) \cdot \sin\left(\frac{\arctan\left(\frac{\epsilon_{\mathrm{im}}}{\epsilon_{\mathrm{re}}}\right)}{2}\right) + \epsilon_{\mathrm{im}}^3 \right] \right\} \quad (S7)$$

$$dv_2(w, \epsilon_{\mathrm{re}}, \epsilon_{\mathrm{im}}, d)$$

$$= \frac{2}{(\epsilon_{\mathrm{re}}^2 + \epsilon_{\mathrm{im}}^2)^{\frac{7}{4}}}$$

$$\cdot \left\{ \sqrt{\epsilon_{\mathrm{re}}^2 + \epsilon_{\mathrm{im}}^2} \cdot \sin^2\left(\frac{\arctan\left(\frac{\epsilon_{\mathrm{im}}}{\epsilon_{\mathrm{re}}}\right)}{2}\right) + \sqrt{\epsilon_{\mathrm{re}}^2 + \epsilon_{\mathrm{im}}^2} \cdot \cos^2\left(\frac{\arctan\left(\frac{\epsilon_{\mathrm{im}}}{\epsilon_{\mathrm{re}}}\right)}{2}\right) + 2 \right.$$

$$\left. \cdot (\epsilon_{\mathrm{re}}^2 + \epsilon_{\mathrm{im}}^2)^{\frac{1}{4}} \cdot \cos\left(\frac{\arctan\left(\frac{\epsilon_{\mathrm{im}}}{\epsilon_{\mathrm{re}}}\right)}{2}\right) + 1 \right\}$$

$$\cdot \left\{ \left(\epsilon_{\mathrm{im}}^3 + \epsilon_{\mathrm{re}}^2 \epsilon_{\mathrm{im}}\right) \cdot \cos\left(\frac{\arctan\left(\frac{\epsilon_{\mathrm{im}}}{\epsilon_{\mathrm{re}}}\right)}{2}\right) + (\epsilon_{\mathrm{re}}^2 + \epsilon_{\mathrm{im}}^2)^{\frac{1}{4}} \cdot \left(\epsilon_{\mathrm{im}}^3 + \epsilon_{\mathrm{re}}^2 \epsilon_{im}\right) \right.$$

$$\cdot \cos^2\left(\frac{\arctan\left(\frac{\epsilon_{\mathrm{im}}}{\epsilon_{\mathrm{re}}}\right)}{2}\right) + \sin\left(\frac{\arctan\left(\frac{\epsilon_{\mathrm{im}}}{\epsilon_{\mathrm{re}}}\right)}{2}\right)$$

$$\cdot \left[-\epsilon_{\mathrm{im}}^2 \epsilon_{\mathrm{re}} + (\epsilon_{\mathrm{re}}^2 + \epsilon_{\mathrm{im}}^2)^{\frac{1}{4}} \cdot \left(\epsilon_{\mathrm{im}}^3 + \epsilon_{re}^2 \epsilon_{im}\right) \cdot \sin\left(\frac{\arctan\left(\frac{\epsilon_{\mathrm{im}}}{\epsilon_{\mathrm{re}}}\right)}{2}\right) \right.$$

$$\left. \left. - \epsilon_{re}^3 \right] \right\} \quad (S8)$$

Similarly, the analytical expressions for the derivatives $\frac{\partial T}{\partial n}$ and $\frac{\partial T}{\partial k}$ of the power transmission coefficient $T(n,k)$ are:

$$\frac{dT}{dn} = \frac{-32 \cdot \exp(-2ak) \cdot \left(n^3 + 2k^2 + n(-1 + k^2)\right)}{(1 + 2n + n^2 + k^2)^3} \quad (S9)$$

$$\frac{dT}{dk} = \frac{-32 \cdot \exp(-2ak) \cdot \left(k(-1 - 2n + n^2 + k^2) + a\left(2n^3 + n^4 + k^2 + 2n(k^2) + k^4 + (n^2)\left(1 + 2(k^2)\right)\right)\right)}{(1 + 2n + n^2 + k^2)^3} \quad (S10)$$

where

$$a = \frac{wd}{27.2114c} \quad (S11)$$

$$n = \mathrm{Re}\left\{\sqrt{\epsilon_{\mathrm{re}} + i\epsilon_{\mathrm{im}}}\right\} \quad (S12)$$

$$k = \mathrm{Im}\left\{\sqrt{\epsilon_{\mathrm{re}} + i\epsilon_{\mathrm{im}}}\right\} \quad (S13)$$

Supplementary References

(1) Jung, M.-H. Exploration of Two-Dimensional Perovskites Incorporating Methylammonium for High Performance Solar Cells. *CrystEngComm* **2021**, *23* (5), 1181–1200. https://doi.org/10.1039/D0CE01469A.
